# Using mixture modeling to examine differences in perceptual decision-making as a function of the time and method of participant recruitment

**DOI:** 10.3758/s13428-023-02142-0

**Published:** 2023-07-18

**Authors:** Timothy Ballard, Nathan J. Evans, Gina Fisher, David K. Sewell

**Affiliations:** The University of Queensland, St Lucia, Australia

**Keywords:** Perceptual decision-making, Response time modeling, Mixture modeling, Evidence accumulation, Participant recruitment, Amazon Mechanical Turk

## Abstract

We examine whether perceptual decision-making differs as a function of the time in the academic term and whether the participant is an undergraduate participating for course credit, a paid in-person participant, or a paid online participant recruited via Amazon Mechanical Turk. We use a mixture modeling approach within an evidence accumulation framework that separates stimulus-driven responses from contaminant responses, allowing us to distinguish between performance when a participant is engaged in the task and the consistency in this task focus. We first report a survey showing cognitive psychologists expect performance and response caution to be lower among undergraduate participants recruited at the end of the academic term compared to those recruited near the start, and highest among paid in-person participants. The findings from two experiments using common paradigms revealed very little evidence of time-of-semester effects among course credit participants on accuracy, response time, efficiency of information processing (when engaged in the task), caution, and non-decision time, or consistency in task focus. However, paid in-person participants did tend to be more accurate than the other two groups. Groups showed similar effects of speed/accuracy emphasis on response caution and of discrimination difficulty on information processing efficiency, but the effect of speed/accuracy emphasis on information processing efficiency was less consistent among groups. We conclude that online crowdsourcing platforms can provide quality perceptual decision-making data, but recommend that mixture modeling be used to adequately account for data generated by processes other than the psychological phenomena under investigation.

Online crowdsourcing platforms have revolutionized the ways in which psychological scientists collect data. Across the various sub-disciplines in psychology, paid samples from platforms such as Amazon Mechanical Turk, Prolific Academic, or Crowdflower are increasingly being used alongside convenience samples of undergraduate psychology students who provide data in exchange for course credit. For many researchers, crowdsourcing platforms appear to have become the recruitment method of choice, particularly in the wake of calls, spurred by the so-called “reproducibility crisis,” emphasizing the importance of recruiting large numbers of participants (e.g., Benjamin et al., 2018; Schweizer & Furley, 2016; Wicherts et al., 2016; though see Smith & Little, 2018, for an alternative perspective). Restricted capacity to recruit participants for in-lab study in light of the COVID-19 pandemic has also made the move to online platforms a necessity for many.

**Fig. 1:**
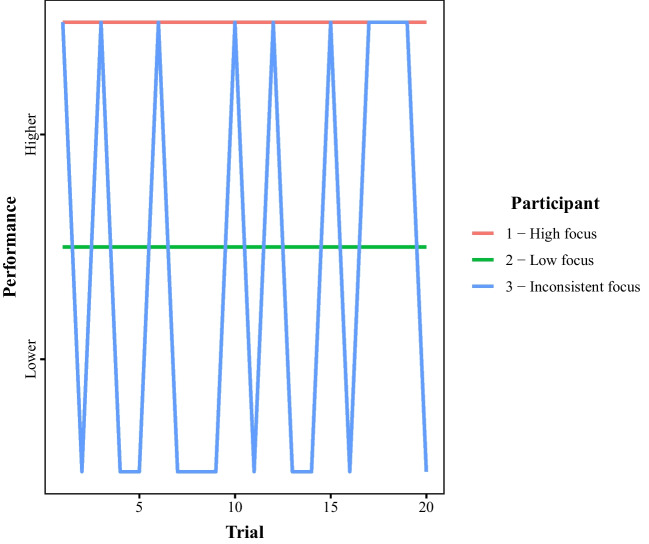
Three participants with different levels of task focus in a hypothetical experiment

Various concerns have been voiced about both crowdsourcing platforms and undergraduate participant pools in regards to the quality of sample data as well as generalizability. For example, it has been argued that participants recruited from crowdsourcing platforms pay less attention to study materials (Goodman et al., 2013; Crump et al., 2013), are more likely to provide invalid responses (Chmielewski & Kucker, 2019), and are more strongly motivated by financial compensation than participants from university samples (Behrend et al., 2011). Meanwhile, samples of undergraduate students have been criticised for being more homogeneous than the general population, raising their own questions about generalizability (Henrich et al., 2010; Roulin, 2015; Miller et al., 2017). It has also been argued that the data obtained from undergraduate students might differ over the course of an academic term (Chan et al., 2017; Porfido et al., 2020; Ebersole et al., 2016; Ratcliff et al., 2001). This argument reflects the seemingly common perception that students who put off enrolling in a study may be less motivated or conscientious, and therefore less engaged with the task. An alternative to online and undergraduate samples is to collect in-lab data from paid participants who are members of the local community. These samples may include high numbers of undergraduates, but are likely to represent a broader range of majors than samples of psychology students. To our knowledge, differences between paid and unpaid (i.e., course credit) university samples has received far less attention.

There are a several ways that the performance of these participant groups could differ from one another. For instance, paid in-lab participants might put more effort into the task than unpaid and/or crowdsourced participants, leading to better overall performance. In the context of crowdsourced samples, financial incentives increase both task engagement and effort (Liang & Hogarth, 2018). However, paid participants do not always outperform unpaid participants, nor do monetary incentives scale monotonically with performance (e.g., Camerer et al., 1999; Gneezy & Rustichini, 2000). Paid participants may approach the task differently if they are more worried about making mistakes than unpaid and/or crowdsourced participants. Monetary reward can counterintuitively produce poorer task performance by diverting attention away from the task at hand in favor of performance monitoring (Ballard et al., 2019; Baumeister, 1984; Markman et al., 2006; Worthy et al., 2009). Furthermore, these participants may be more likely to use strategies that minimize the risk of errors (Koriat & Goldsmith, 1994). Moreover, if unpaid and crowdsourced participants do put in less effort than paid in-lab participant, this could manifest in different ways (see Fig. 1). Unpaid and crowdsourced participants may simply demonstrate a consistently lower level of focus throughout an experiment (e.g., Participant 2 in Fig. 1), leading to a moderate level of performance. Alternatively, they may be able to achieve similar levels of performance when focused on the task as their paid in-lab counterparts, but they may do so less consistently (e.g., Participant 3 in 1). For example, unpaid and crowdsourced participants may be more prone to lapses in attention – particularly the crowdsourced participants completing the study online in a location of their choice – meaning that their performance is only worse on some portion of trials. Importantly, there are several reasons and ways that paid, unpaid, and crowdsourced participants could differ from one another in how they complete psychological experiments; the participants’ performance level when engaged in the task, the consistency of participants’ task engagement, and their level of task caution.

Within the area of speeded decision-making, model-based approaches have been developed to separate task performance from response caution. Specifically, evidence accumulation models have become the dominant framework for explaining how quick decisions about simple stimuli are made. According to these models, participants accumulate evidence for each decision alternative at some rate (known as the drift rate) until the evidence for one alternative reaches a specified level of evidence (known as the decision threshold), triggering a response. Importantly, these models are able to decompose the response made on each trial, and the time taken to make it, into separate cognitive constructs that govern the proposed underlying decision-making process. This allows evidence accumulation models to separate the constructs such as task performance – reflected in the estimated drift rate – from task caution – reflected in the estimated threshold – that are conflated in assessments of observed variables alone (e.g., response choice or response time). Importantly, evidence accumulation models provide an approach for separating some of the different ways that paid, unpaid, and crowdsourced participants could differ from one another. Furthermore, investigating the impact of different recruitment methods seems particularly important within speeded decision-making, as the use of online platforms such as Mechanical Turk for data collection has become more common within this area. As far as we are aware, no systematic investigation into how the decision processes of participants recruited from these platforms – or the lack of precise experimental control provided by online environments – might differ from those of in-lab participants who perform these tasks under more controlled conditions.

However, even these complex model-based approaches are typically unable to explicitly separate poor performance caused by an overall lower level of task focus from poor performance on specific trials due to inconsistency in task focus (e.g., the presence of attention lapses). Specifically, these models typically assume that all responses are the result of the psychological process of interest (e.g., a stimulus-driven evidence accumulation process). Similar assumptions are made in standard analyses of response time and accuracy (e.g., approaches based on signal detection theory). In order to ensure that the results are not influenced by data generated by processes other than the psychological process of interest, researchers typically try to find and exclude these contaminant responses using heuristics of what we believe these contaminants might look like. For example, researchers might exclude participants if they produce long strings of the same response or reliably alternate between two responses (e.g., switching back and forth between ‘left’ versus ‘right’; see Curran (2016) for review). At the level of the individual trial, researchers in speeded decision-making will often exclude fast responses that are suspected to be the result of fast guessing, slow responses that are suspected to be the result of attentional lapses, and participants who have low accuracy that are suspected to generally be doing the task incorrectly or inattentively. In some cases, these contaminants are easy to detect, as these data are completely separate from what is possible from data generated by the process of interest. For instance, responses that are faster than human limits on perceptual processing could only be the result of something outside of the decision-making process of interest (i.e., guesses).

However, in most cases, there is considerable overlap between the ranges of data that could be generated by the psychological process of interest and the data that could be generated by a different, contaminant process. For instance, responses that are quick, but not too quick to be impossible, could be fast guesses (i.e., contaminants), or could instead be stimulus-driven decisions (i.e., data of interest). This creates an implicit trade-off for researchers between basing inferences on as much of data as possible versus avoiding the influence of contaminant responses. On one hand, if researchers set very conservative boundaries for excluding contaminants, then their assessments may be contaminated by trials that are not generated by the process of interest, which may lead to conclusions that do not accurately reflect the process that they claim to be about. On the other hand, if researchers set very liberal boundaries for excluding contaminants, then their assessments may throw away a large subset of trials – likely from a specific region of the data space – that reflect the process of interest, which could bias conclusions based on which trials remain.

One attempt to address this tradeoff in the context of evidence accumulation models was proposed by Ratcliff & Tuerlinckx (2002), who modeled choice and response time data assuming it comprised a mixture of stimulus-driven decisions produced by a diffusion decision process and a uniform distribution of response times from contaminant responses. This approach allows for fairly broad coverage of contaminants, as they are assumed to be distributed across the entire range of observed response times with rates proportional to the observed rates of correct and error responses. A limitation of this approach, however, is that it is indifferent to the nature of the contaminant responses, and so the assumed uniform distribution of contaminants may differ from the distribution of responses generated from a specific kind of contaminant process. For example, responses produced by a guessing process will be evenly distributed across correct and error responses rather than appearing in the same proportions as correct and error responses arising from stimulus-driven decisions. Similarly, the distribution of response times generated by guesses will be right-skewed if they are the result of evidence accumulation in the absence of stimulus information, rather than uniformly distributed.

Here, we argue that researchers do not need to implicitly trade-off between the potential for excluding data that are generated by the process of interest and the potential for including data that are generated by a different process. Instead, we propose a different approach, where the proposed process also includes explicit explanations for how these contaminants might occur. Specifically, within the context of separating poor overall performance due to low task focus from inconsistency in focus due to attention lapses, we propose using a mixture modelling approach, where trials can either be the result of stimulus-driven decisions, or participants not paying attention to the stimulus and making uninformed choices. Importantly, this provides a method for estimating how good people are at the task when they are paying attention, and how often people ignore the stimulus and make random responses either because of fast guessing or attention lapses, separating the two different ways that participants recruited through different methods might differ in task performance.

Our proposed mixture modelling approach extends similar methods of accounting for contaminant data that have been used in the visual working memory literature. To accurately measure storage capacity limits, it is vital to explicitly model guesses due to attention lapses to avoid underestimation of performance (e.g., Rouder et al., 2008). The approach has been extended in the visual working memory domain to assessments of representational precision in memory (e.g., Bays et al., 2009), and has also been incorporated into assessments of visual working memory using evidence accumulation models (Donkin et al., 2013; Lilburn & Smith, 2020; Sewell et al., 2016; Smith et al., 2016). In all cases, explicit modeling of guesses is required to ensure accurate measurement of the memory processes under investigation. Applying guessing-mixture models to task domains that sit outside the memory domain is critical for assessing the benefits of the approach to other situations where inattention has the potential to compromise the integrity of behavioral data and create a misleading picture of processes underpinning performance.

In this paper, we use the approach above to examine whether perceptual decision-making processes differ as a function of the time and method of recruitment. We argue that these factors are perceived as likely to relate to participant levels of and consistency in task focus, and create an ideal setting to evaluate the general utility of the mixture modeling approach. In the sections that follow, we report three preregistered studies. The first briefly summarizes the results of a survey of cognitive psychologists that we conducted in order to understand researchers’ intuitions regarding possible differences across participant groups. We then report two experiments that examine the extent to which these intuitions are reflected in participant data. These studies use common perceptual decision-making paradigms to examine whether the effects of standard experimental manipulations of discrimination difficulty and speed/accuracy emphasis on performance when engaged in the task, the consistency in this task focus across trials, response caution, and the time taken for non-decision processes (stimulus encoding and motor responses) differ among participants recruited at different times (i.e., early versus late in semester) and in different ways (i.e., undergraduates participating in the lab for course credit, community members participating in the lab for payment, and paid participants recruited via Mechanical Turk). Experiment 1 examines these effects using a random-dot motion discrimination task whereas Experiment 2 uses a brightness discrimination task.

Our mixture modelling approach involves using the Linear Ballistic Accumulator (LBA) model (Brown & Heathcote, 2008) in the context of model application (Crüwell et al., 2019) in order to better understand the differences between groups in terms of the overall levels of latent variables that unpin the decision-making process, and how these variables are affected by standard experimental manipulations. The LBA assumes that the evidence for each response alternative accumulates independently. There were two response alternatives in each experiment, so the model included two evidence accumulators. The amount of evidence for each alternative at the start of each decision trial is drawn from a uniform distribution [0, *A*]. The evidence then accumulates linearly. For each accumulator, the rate at which evidence accumulates (the drift rate) is drawn from a normal distribution with mean *v* and standard deviation *sv*. Evidence accumulates until the evidence for one alternative breaches a threshold (*b*), at which point that alternative is selected and the response is made.

The difference in the rates of evidence accumulation for the correct and incorrect response alternatives captures the efficiency of information processing. Differences in this value across participant groups captures differences in overall levels of performance. Aside from differing in overall level, it is plausible that groups differ in how their efficiency of information processing changes as a function of common experimental manipulations such as speed/accuracy emphasis or discrimination difficulty. For example, it is possible that the information processing among one participant group is more sensitive to how difficult a stimulus is to discriminate than the information processing among other groups. The response threshold quantifies the degree of caution. Higher threshold reflect more caution as they require more evidence before a decision is made. The LBA also includes a non-decision time parameter ($t_0$) that captures the component of the response time attributable to processes other than the decision process (e.g., encoding the stimulus and manually executing the response). Response caution and non-decision time may also vary across groups in terms of their overall level and in terms of how they respond to the manipulation of speed/accuracy emphasis.

To develop the mixture model, we incorporated an additional parameter, $p_{guess}$, which was the probability of the $v_{diff}$ for any given trial being 0, which would reflect the participant failing to attend to the stimulus in that trial, and therefore, receiving no useful information about the stimulus. Therefore, this parameter provides an indication of the proportion of trials where a participant was not using the stimulus to guide their decision making, where lower values reflect more stimulus-driven decisions. In other words, $p_{guess}$ quantifies the (in)consistency in task focus for each participant. Because the drift rate for both accumulators on guess trials is the same, the model assumes that guess responses tend to be slower than stimulus-driven responses^1^. In principle, one could allow the guess accumulation rates to be faster or slower than the mean of the stimulus-driven rates. This might be useful in situations where guesses are systematically faster (or even more slow than assumed by this model). For simplicity, however, here we use a model that assumes the mean rate of evidence accumulation is the same for guesses and stimulus-driven driven responses.

To provide context for our analysis, we use the mixture model to answer the following three research questions:

1) How do method and time of recruitment relate to the proportion of guess trials, efficiency of information processing, response caution, or non-decision time?

2) How do method and time of recruitment relate to the effects of speed/accuracy emphasis on efficiency of information processing, response caution, and non-decision time?

3) How do method and time of recruitment relate to the effects of discrimination difficulty on the efficiency of information processing?

All studies were approved by The University of Queensland Health and Behavioural Sciences ethics committee (approval number 2017001346). The data and code necessary to conduct all the analyses presented in this paper, as well as the source code used to generate the experimental stimuli, are publicly available and can be found at https://osf.io/qgzfw/. All studies presented in this paper were preregistered prior to the research^2^. The preregistrations can be found at https://osf.io/rax2b.

## Survey

In this section, we summarize the results of a brief survey we conducted to examine expectations among cognitive psychologists regarding how different participant groups would perform. The full details of this study are reported in the supplementary materials. We presented 51 cognitive psychologist with two scenarios describing hypothetical experiments with participants recruited at different times in the semester. In the *early* condition, respondents were asked to “Imagine you are planning to run a basic cognitive experiment involving speeded choice using a sample of participants recruited within the FIRST three weeks of semester.” In the *late* condition, the description was identical except that the hypothetical participants were recruited “within the FINAL three weeks of semester”.

For each scenario, participants rated expected performance and response caution for three different participant groups: 1) Undergraduate students participating in exchange for course credit and who do not receive financial remuneration (*local, credit* condition), 2) Members of the community participating in exchange for financial compensation and remunerated at a rate that is standard for the department (*local, paid*), and 3) Participants recruited via online crowd sourcing platforms (e.g., Amazon Mechanical Turk) and remunerated at a rate that is standard for the department (*online, paid*).

Expected performance was measured by asking respondents to indicate how they expected participants to perform on the task on a 7-point scale ranging from “extremely poorly” to “extremely well”. Expected caution was measured by asking respondents to indicate how cautiously they expected the hypothetical participants to respond, with low caution meaning sacrificing accuracy in order to respond quickly and high caution meaning sacrificing speed in order to respond accurately. Caution was rated on a 7-point scale ranging from “extremely low caution” to “extremely high caution”.

**Fig. 2:**
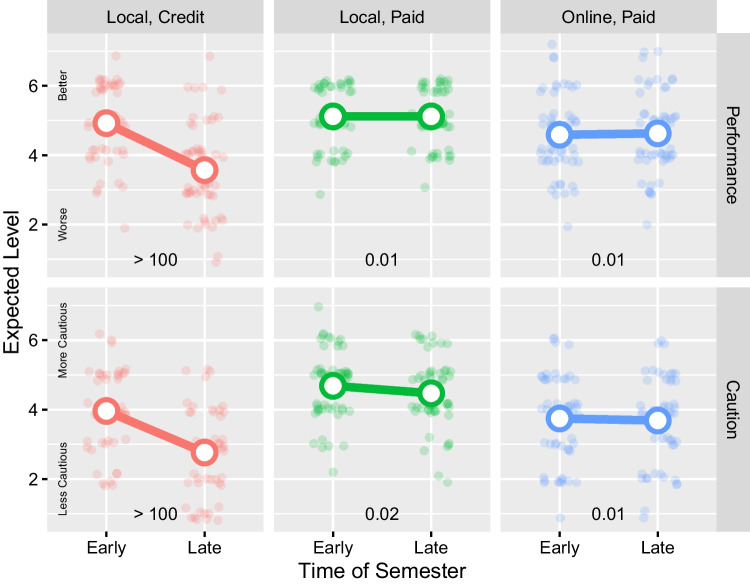
Effects of time of semester and population on expected performance and response caution in a hypothetical speeded choice experiment. Lines represent the condition means, whereas points represent the individual ratings from each participant. A small amount of jitter was added to the points so that the relatively frequency of responses can be seen more clearly

We examined the effects of time of semester and participant population on expected performance and response caution using a pair of Bayesian mixed models which were implemented via the brms package (Bürkner, 2017) in R. The first model examined the effects of these variables on expected performance, and the second examined the effects on expected response caution. We modeled the effects of participant population by including three dummy-coded variables and forcing the model intercept to equal zero. The first dummy-coded variable had a value of 1 for the local, credit group and a value of 0 for the local, paid and online, paid groups. The second had a value of 1 for the local, paid group and 0 for the local, credit and online, paid groups. The third had a value of 1 for the online, paid group and 0 for the local, credit and local, paid groups. The coefficients associated with these variables represent the average level of the dependent variable in each group. The time of semester variable was coded such that it had a value of -1 for the participants recruited in the first 3 weeks of semester, and 1 for the participants recruited in the final 3 weeks of semester. Each model contained the three dummy-coded participant group variables and the three two-way participant population $\times$ time of semester interactions. This analysis allowed us to examine the overall effects of recruitment method as well as the effects of time of semester within each participant population (indexed by the two-way interactions). Each model also included the random effect of participant.

A summary of the results are presented in Fig. 2. Full statistical analyses are presented in the supplementary material. To summarize, the cognitive psychologists who responded to our survey expected task performance (Bayes Factor> 100, 95% credible interval = (-0.85, -0.50)) and response caution (BF> 100, CI = (-0.80, -0.41)) in a speeded choice experiment to be lower for a sample of undergraduates recruited later in semester compared to a sample recruited earlier in semester. This result is consistent with the perception that undergraduates recruited at different times in the semester are expected to perform differently on average; perhaps due to differences in task focus, engagement, or overall motivation. This time-of-semester effect was not expected by the cognitive psychologists to emerge for paid participants (for task performance difference among local, paid: BF = 0.01, CI = (-0.18, 0.19); for caution difference among local, paid: BF = 0.02, CI = (-0.30, 0.09); for task performance difference among online, paid: BF = 0.01; CI = (-0.15, 0.20); for caution difference among online, paid: BF = 0.01, CI = (-0.23, 0.17). The cognitive psychologists also expected the overall levels of both variables to be higher among local, paid participants than local, credit participants (local, paid minus local, credit task performance: BF> 100; CI = (0.62, 1.13); caution: BF> 100; CI = (0.94, 1.50)) and online, paid participants (local, paid minus online, paid task performance: BF> 100, CI = (0.25, 0.76); caution: BF > 100; CI = (0.58; 1.14)). The results of the survey establish an expected benchmark against which we compare actual participant data.

## Experiments 1 and 2

In this section, we report two experiments that test the intuitions that were clarified by the survey above and demonstrate how the mixture modeling approach described above can improve the insights achieved when data from these different participant groups are analyzed. Experiment 1 uses a random dot motion discrimination paradigm and Experiment 2 uses a brightness discrimination paradigm. The logical structure of the two experiments is otherwise identical (e.g., both studies manipulate difficulty by varying the perceptual properties of the stimulus and use instructions to manipulate speed/accuracy emphasis). To facilitate interpretation of the results, we report the two experiments together.

**Table 1:** Sample Details for the Motion (Exp 1) and Brightness (Exp 2) Discrimination Experiments

	$N_{total}$	$N_{complete}$	$\%_{complete}$	$\%_{passed}$	$age_{m}$	$age_{sd}$	$\%_{female}$	$\%_{male}$	$\%_{other}$
Experiment 1 (Motion)									
Early									
Local, Credit	42	42	100.0	92.9	20.8	7.0	76.2	23.8	0.0
Local, Paid	32	30	93.8	96.7	22.2	3.4	78.1	21.9	0.0
Online, Paid	75	63	84.0	98.4	37.1	10.5	49.3	49.3	0.0
Late									
Local, Credit	34	34	100.0	100.0	19.9	4.1	76.5	23.5	0.0
Local, Paid	33	32	97.0	96.9	23.9	13.8	69.7	27.3	3.0
Online, Paid	65	60	92.3	98.3	34.4	10.7	30.8	67.7	0.0
Experiment 2 (Brightness)									
Early									
Local, Credit	38	38	100.0	94.7	19.3	2.8	63.2	34.2	0.0
Local, Paid	32	32	100.0	90.6	21.0	2.0	68.8	31.2	0.0
Online, Paid	73	58	79.5	96.6	36.7	11.6	42.5	57.5	0.0
Late									
Local, Credit	32	32	100.0	96.9	19.2	5.2	81.2	18.8	0.0
Local, Paid	31	31	100.0	87.1	21.4	2.5	77.4	22.6	0.0
Online, Paid	70	57	81.4	98.2	35.1	10.4	42.9	57.1	0.0

Note: $N_{total}$, $N_{complete}$, $\%_{complete}$, and $\%_{passed}$ indicate the number of participants who began the experiment, the number of those who completed the experiment, the percentage of participants who completed the experiment, and the percentage of those who passed all five attention checks respectively. $\%_{female}$, $\%_{male}$, $\%_{other}$ indicate the percentage of participants identifying as female, male, or other respectively. Three participants chose not to respond to the gender item. As a result, the percentages across the three gender categories do not all sum to 100. Two participants chose not to report their age

## Method

### Participants

Participants were recruited from three different populations. The first subset of participants consisted of first year psychology students at The University of Queensland (UQ) who participated in exchange for course credit (the *local, credit* group). The second subset consisted of members of the UQ local community who were reimbursed $10 (AUD) for their participation (the *local, paid* group). The third subset was recruited via the Amazon Mechanical Turk online crowdsourcing platform and were reimbursed $4 (USD) for their participation (the *online, paid* group). We restricted the sample to participants in the US with approval rates of at least 95%. Each of these populations were sampled from two different times–once during the first three weeks of the academic semester (the *early* group) and again during the final three weeks of the semester (the *late* group). As a result, there were six different participant groups for each experiment.

Details regarding sample size, completion rates, attention check performance (explained below) and demographics for each participant group are presented in Table 1. As can be seen, more participants dropped out of the experiment in the online, paid group. The online, paid group also tended to be older and included a higher proportion of male participants than either of the local groups. Only participants who completed the full experiment were retained in the analyses. The proportion of participants passing the attention checks was very high across all participant groups. Many have advocated for the removal of participants who fail attention checks on the assumption that doing so reduces noise and increases statistical power (Curran, 2016; Zallot et al., 2021). However, others have argued that doing so can increase the risk of bias in the analysis because participants who fail attention checks tend to differ demographically from participants who do not Anduiza & Galais (2017). The decision to exclude a participant who failed an attention check item also implicitly assumes that all of this participant’s data is contaminated. However, it is plausible that in some proportion of trials, the participant responded attentively. If this is true, then the researcher must discard valid observations. One of the benefits of the mixture modeling approach is that it explicitly accounts for the attentional lapses that attention checks are designed to protect against and disentangles these lapses from the psychological process of interest. As such, using this approach, we avoid the need to filter participants based on attention check performance—and the potential for bias and discarding of valid data that come with it—while simultaneously allowing inferences to be made that are not affected by the presence of contaminant responses.

**Fig. 3:**
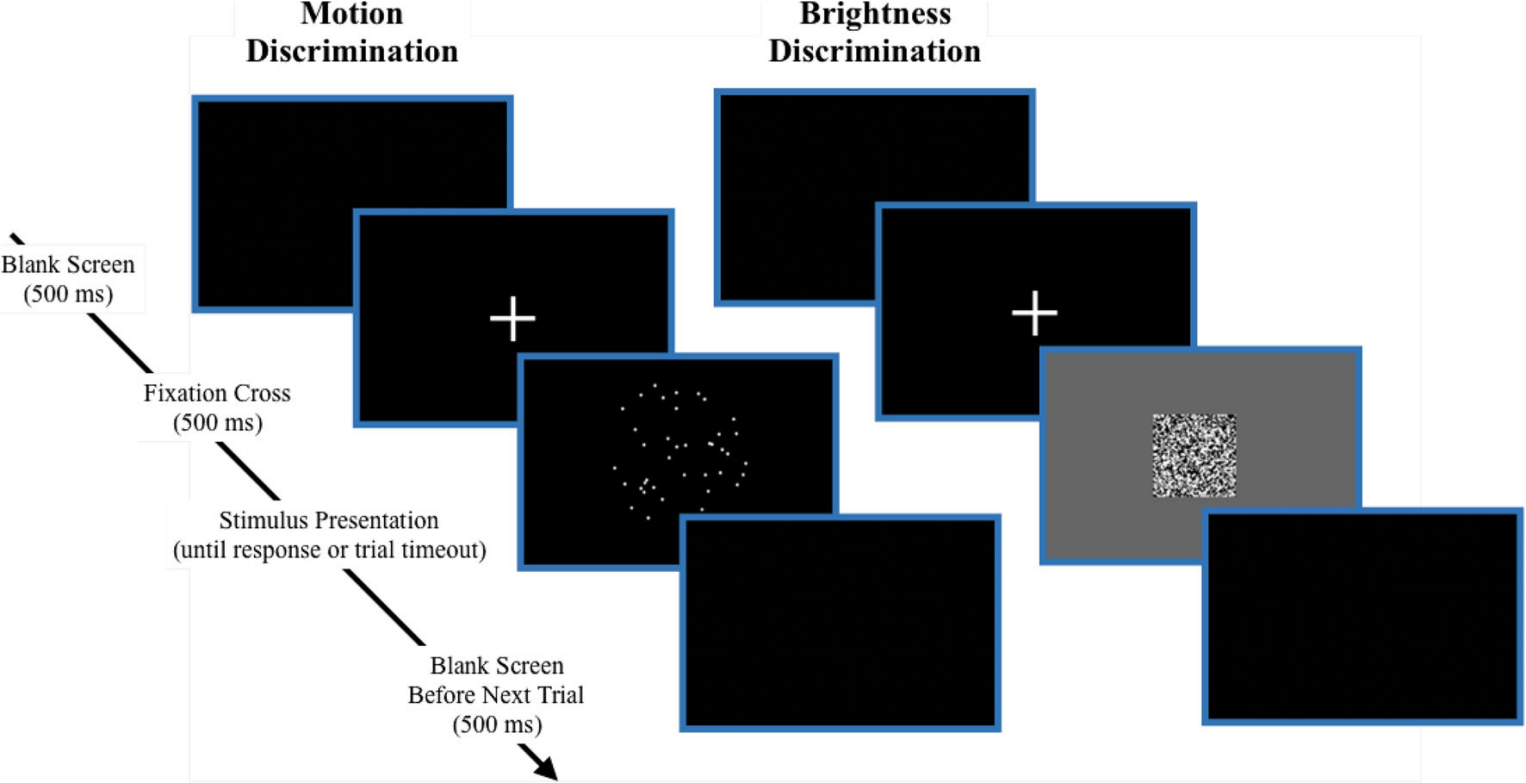
Sequence of experimental trial in the motion and brightness discrimination tasks. In the motion discrimination task (Experiment 1), participants had to identify whether the dots moved mostly left or mostly right. In the brightness discrimination task (Experiment 2), participants had to identify whether the patch contained more white pixels or more black pixels. Note that the stimulus images shown here are not drawn to scale

### Design and stimuli

Each experiment used a 2 (recruitment time: early vs late in semester) $\times$ 3 (participant population: local, credit, local, paid, and online, paid) $\times$ 2 (emphasis: speed vs accuracy) $\times$ 4 (difficulty: very easy, easy, hard, very hard) factorial design. Time of semester and population were between-participant factors, whereas emphasis and difficulty were within-participant factors.

#### Experiment 1: motion discrimination

The participants in Experiment 1 performed a random dot motion discrimination task. In each trial, they were presented with a cloud of 40 moving dots (see Fig. 3). Across successive frames during stimulus presentation, some proportion of the dots moved coherently either toward the left or the right of the viewing aperture, while the other dots were moved in a random direction. The participant’s task was to identify whether the dots were moving mostly to the left (in which case, they responded by pressing the “z” key) or mostly to the right (“/” key response). Discrimination difficulty was manipulated by varying the proportion of the dots moving coherently (very easy: 0.25, easy: 0.20, hard: 0.15, very hard: 0.10).

The stimulus was generated using a version of the “white noise” algorithm (Pilly & Seitz, 2009). The diameter of the cloud stimulus was approximately 33% of the screen height. Each dot had a diameter of approximately 0.66% of the screen height. The dot positions were updated approximately every 16.6 ms. On each update, dots were independently selected according to the trial coherence level to move coherently or randomly on the next update. Coherent dots moved at a rate such that it would take a single dot 3000 ms to traverse the entire cloud. If the new location would be outside the cloud, the dot was repositioned randomly. Non-coherent dots were repositioned randomly.

#### Experiment 2: brightness discrimination

The participants in Experiment 2 performed a brightness discrimination task. In each trial, they were presented with a static 64 x 64 square of black and white pixels on a 320 x 200 pixel grey background. On each trial, the program randomly allocated some proportion of the pixels in the square to be either white or black. The participant’s task was to identify whether the patch was dark, where there are more black pixels than white (“z” key response); or light, where there are more white pixels than black (“/” key response). Difficulty was manipulated by varying the proportion of pixels that the dominant color occupied (very easy: 0.54, easy: 0.53, hard: 0.52, very hard: 0.51).

### Procedure

Participants began by completing 10 practice trials (which were not analyzed). Each experiment was then broken down into 4 blocks of 200 trials each. Prior to each block, participants completed an attention check item in which they were asked to select a particular number from a list. Speed versus accuracy emphasis was manipulated between blocks. In speed blocks, participants were instructed to respond as quickly as possible. In this condition, the participant had to respond within 1 second. If the participant did not make a response within 1 second, a “TOO SLOW” message appeared and the trial was recorded as a non-response. In accuracy blocks, participants were instructed to respond as accurately as possible. In this condition, they had 3 seconds to respond. Speed and accuracy blocks were presented in alternating order, with the first block being randomly determined.

For each unique combination of the difficulty $\times$ speed/ accuracy emphasis manipulation, each stimulus was presented the same number of times. That is, in Experiment 1, half of the stimuli in each cell of the design displayed dots moving to the left and half displayed dots moving to the right. Likewise in Experiment 2, half of the stimuli in each cell displayed patches with more black pixels and half displayed patches with more white pixels. Difficulty was manipulated within blocks. The order of stimuli was randomized within each block. Outside of the practice trials, participants did not receive any feedback on their decisions.

### Data analysis

We used Bayes factors (BFs) to evaluate the evidence for the effects reported below, which represent the ratio of evidence for two hypotheses. Bayes factors greater than one indicate evidence for an effect/difference whereas Bayes factors less than one indicate evidence for the absence of an effect/difference. We use the classification scheme reported by Lee & Wagenmakers (2013) to describe the strength of evidence. Under this scheme, Bayes factors between 3 and 10 (or between 1/10 and 1/3) correspond to “moderate” evidence, Bayes factors between 10 and 30 (or between 1/30 and 1/10) correspond to “strong” evidence, Bayes factors between 30 and 100 (or between 1/100 and 1/30) correspond to “very strong” evidence, and Bayes factors greater than 100 (or less than 1/100) correspond to “extreme” evidence. For simplicity, we report Bayes factors corresponding to extreme evidence as “$> 100$” or “$< 0.01$”. We also use 95% credible intervals (CI) to summarize the magnitude of effects.

The analyses of accuracy and response time were conducted via a series of Bayesian mixed models using the *BayesFactor* package in R. For each study, we examined the effects of participant population, time of semester, speed/accuracy emphasis, and difficulty on the proportion correct and mean response time. Each model included the random effect of participant. The Bayes factors were obtained using a top-down model comparison approach. The numerator in each comparison was the full model which contained all four variables and all possible interactions and the denominator was a model with the relevant effect removed. Thus, Bayes factors greater than one indicated evidence for an effect whereas Bayes factors less than one indicated evidence for the absence of an effect.

The Bayes factors pertaining to the LBA model parameters were computed using the Savage-Dickey density ratio method (Wagenmakers et al., 2010), comparing the evidence for a difference between conditions (or from zero) with a null model which assumed no difference. Across the two studies, the number of decision trials encountered by each participant group ranged from 24000 to 50400, with each participant contributing 800 trials. This is consistent with the recommended sample size for accurate parameter estimation using the LBA (e.g., Donkin, 2009). Further details regarding parameter estimation are provided below.

## Results

### Behavioral results

#### Accuracy

**Fig. 4:**
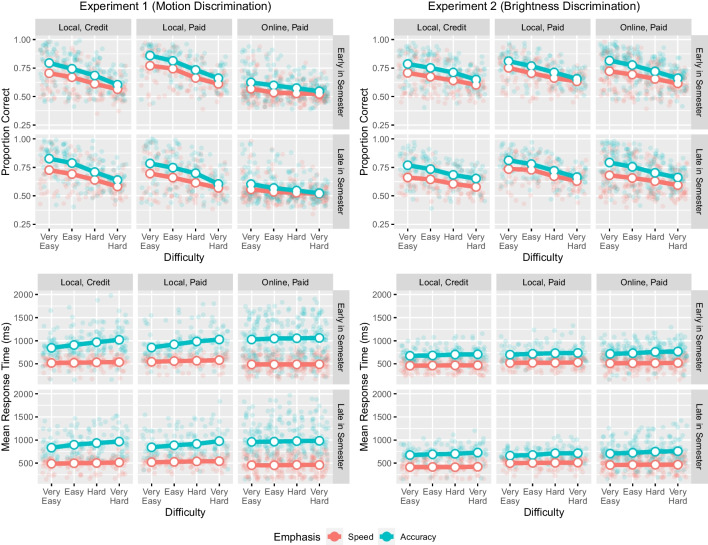
Effects of participant population, time of semester, speed/accuracy emphasis, and difficulty on accuracy (top row) and response time (bottom row) in the motion discrimination (left column) and brightness discrimination (right column) tasks. Lines represent the condition means, whereas points represent the participant means

The results of the accuracy and response time analyses are presented in Fig. 4 and Table 2. The analyses revealed extreme evidence for the effect of participant population on accuracy in both studies. In both experiments, accuracy was higher in the local, paid group than in the other two groups (motion discrimination: local, paid minus local, credit = (0.002, 0.029); local, paid minus online, paid = (0.135, 0.160); brightness discrimination: local, paid minus local, credit = (0.023, 0.049); local, paid minus online, paid = (0.007, 0.031)). In the motion discrimination task, the local, credit participants were more accurate than online, paid participants (local, credit minus online, paid = (0.120, 0.143)), whereas in the brightness discrimination task, the online, paid participants were more accurate than the local, credit group (local, credit minus online, paid = (-0.029, -0.005). We also found extreme evidence for the effect of emphasis and difficulty on accuracy in each study, with the proportion correct increasing under accuracy emphasis (accuracy minus speed in motion discrimination = (0.050, 0.071); in brightness discrimination = (0.059, 0.079) and decreasing in response to more difficult stimuli (in motion discrimination = (-0.048, -0.038); in brightness = (-0.045, -0.035). The main effects of time of semester on accuracy were mixed. There was moderate evidence for an effect in the motion discrimination task, with accuracy being higher for participants recruited earlier in the semester (early minus late = (0.005, 0.026)), but the evidence for this difference in the brightness discrimination task (0.004, 0.024) was weaker.

The results for interactions differed between the two studies, particularly for interactions involving participant population. There was extreme evidence for a participant population $\times$ time of semester interaction in the motion discrimination task. In this task, local, credit participants were actually more accurate when completing the study later in semester compared to earlier (early minus late = -0.045, -0.009), whereas the paid participants were more accurate when recruited earlier in semester (among local, paid = (0.039, 0.079); among online, paid = (0.0005, 0.0289)). There was moderate evidence against the participant population $\times$ time of semester interaction in the brightness discrimination task.

There was moderate evidence for an emphasis $\times$ participant population interaction in the motion discrimination task, with the accuracy improvement when accuracy was emphasized being higher among local, credit (accuracy minus speed = (0.055, 0.092)) and local, paid (0.050, 0.090) participants than for online, paid participants (0.023, 0.051). There was moderate evidence against this interaction in the brightness discrimination task. Finally, there was extreme evidence for a difficulty $\times$ population interaction in the motion discrimination task, with the effect of difficulty on accuracy being stronger among local, credit (-0.062, -0.046) and local, paid (-0,063, -0.045) participants than online, paid participants (-0.028, -0.015). There was extreme evidence against this interaction in the brightness discrimination task. These three interactions converged on the notion that, in the motion discrimination task, the effects of emphasis, difficulty, and time of semester on accuracy were weaker for the online, paid participant group, as this group was less accurate overall. However, this was not the case in the brightness discrimination task. Both studies provided at least moderate evidence against all other interactions.

**Table 2:** Bayes Factor Results for the Effects of Participant Population, Time of Semester, Speed/Accuracy Emphasis, and Difficulty on Accuracy and Mean Response Times

	Accuracy		Mean Response Time	
	Motion	Brightness	Motion	Brightness
Time of Semester	3.48	2.03	4.62	5.47
Population	$> 100$	$> 100$	0.02	$> 100$
Emphasis	$> 100$	$> 100$	$> 100$	$> 100$
Difficulty	$> 100$	$> 100$	1.19	0.04
Population $\times$ Time	$> 100$	0.29	0.02	0.01
Emphasis $\times$ Time	0.07	0.17	0.09	0.42
Emphasis $\times$ Population	6.02	0.28	$> 100$	3.54
Difficulty $\times$ Time	$< 0.01$	$< 0.01$	$< 0.01$	$< 0.01$
Difficulty $\times$ Population	$> 100$	$< 0.01$	$< 0.01$	$< 0.01$
Difficulty $\times$ Emphasis	0.27	0.16	0.09	0.02
Emphasis $\times$ Population $\times$ Time	0.06	0.03	0.03	0.11
Difficulty $\times$ Population $\times$ Time	$< 0.01$	$< 0.01$	$< 0.01$	$< 0.01$
Difficulty $\times$ Emphasis $\times$ Time	$< 0.01$	0.01	$< 0.01$	$< 0.01$
Difficulty $\times$ Emphasis $\times$ Population	$< 0.01$	$< 0.01$	$< 0.01$	$< 0.01$
Difficulty $\times$ Emphasis $\times$ Population $\times$ Time	$< 0.01$	$< 0.01$	$< 0.01$	1.65

Note: The BFs were obtained using a top-down model comparison approach. The numerator in each comparison was the full model and the denominator was a model with the relevant effect removed. Thus, BFs > 1 indicate evidence in support of the effect, whereas BFs < 1 indicate evidence against it

#### Mean response time

The analyses revealed extreme evidence for the effect of emphasis on mean response time in both studies, with response times being higher when accuracy was emphasized than when speed was emphasized (accuracy minus speed in motion discrimination = (415.86, 465.49); in brightness discrimination = (215.67, 247.37). There was extreme evidence for an effect of participant population on response time in the brightness discrimination task, with both groups of paid participants taking longer to respond on average than local, credit participants (local, paid minus local, credit = (21.32, 62.93); online, paid minus local, credit = (27.23, 64.37)); local, paid minus online, paid = (-23.02, 15.30). By contrast, there was very strong evidence for the absence of an effect in the motion discrimination task. There was strong evidence against an effect of difficulty on response time in the brightness discrimination task, but the evidence for/against this effect in the motion discrimination task was non-diagnostic. Finally, there was moderate evidence for an effect of time of semester on response time in both experiments, with participants recruited earlier in semester taking longer to respond (in motion discrimination = (13.69, 62.69); in brightness discrimination = (8.41, 39.79)).

There was extreme evidence for an emphasis $\times$ participant population interaction on response time in the motion discrimination task. In this task, the effects of speed/accuracy emphasis were stronger for the online, paid group (accuracy minus speed = (501.02, 569.15)) than the local, credit (363.79, 452.13) or local, paid (331.49, 428.03) groups. There was moderate evidence for this interaction in the brightness discrimination task. In the brightness task, the effects of speed/accuracy emphasis were stronger in both the online, paid (226.44, 271.05) and local, credit (225.01, 281.70) groups than in the local, paid group (162.25, 221.79). Both studies provided at least moderate evidence against all other interactions, with the exception of the four-way interaction in the brightness experiment, for which the evidence was non-diagnostic.

### Computational modeling results

We analyzed each participant group using separate models. We constrained all parameters except *v* to have the same value across accumulators, meaning that our model had 5 main parameters: *A*, *B*, $t_0$, $v_{mean}$ (the average drift rate across the two accumulators), and $v_{diff}$ (the difference in drift rate between the two accumulators), with *sv* fixed to 1 to solve a scaling issue within the model. As is common practice (e.g., Brown & Heathcote, 2008), we express threshold in terms of the difference between the raw threshold and the maximum starting point for evidence (denoted *B*, where $B=b-A$). For each individual, we estimated the mean over conditions for each of these five parameters, as well as the difference between emphasis conditions in *B*, $t_0$, and $v_{diff}$, and a parameter for the linear difference between difficulty conditions in $v_{diff}$ (i.e., difficulty conditions dummy-coded as -1.5, -0.5, 0.5, and 1.5). Importantly, we constrained $v_{mean}$ to take the same value across conditions, to prevent an identifiability issue within the LBA (Evans, 2020). This meant that each individual had 10 estimated parameters: 5 mean parameters, 4 differences parameters, and the mixture parameter $p_{guess}$ (though note that as *A* is not of theoretical interest within the current study, we do not discuss it further).

We also used a Bayesian hierarchical implementation that constrained the parameters of each individual to follow group-level distributions, allowing participants to mutually inform the estimation of processes of one another via the group-level. Most importantly, the hierarchical model provides group-level estimates for the parameters of interest, allowing us to directly estimate – with appropriate uncertainty via the posterior distributions – these parameters for each group of participants. Specifically, we constrained the individual estimates of all of the 5 mean parameters to follow positively truncated normal distributions, the 4 difference parameters to follow normal distributions, and the mixture parameter to follow a truncated normal distribution between 0 and 1. This meant that each parameter in the model had two hyperparameters: the group-level mean ($\mu$) and the group-level standard deviation ($\sigma$). The priors on the group-level mean hyperparameters for all of the standard parameters can be seen in Table 3; the priors on all group-level mean hyperparameters for changes across conditions were *N*(0, 0.3), and the priors on all group-level standard deviation hyperparameters were $\Gamma (1,1)$^3^.

**Table 3:** Priors on the Population Mean Distributions Used in the LBA Analysis for Experiments 1 and 2

Parameter	Mean	SD	Lower	Upper
*A*	1	0.5	0	$\infty$
*B*	1	0.5	0	$\infty$
$t_0$	0.3	0.1	0	$\infty$
$v_{mean}$	3	1	0	$\infty$
$v_{diff}$	1	1	0	$\infty$
$p_{guess}$	0.5	0.2	0	1

Prior to investigating the research questions, we first examined the ability of the mixture model to explain the data, relative to a standard implementation of the LBA. To do this, we calculated the deviance information criterion (DIC) for the mixture model and a standard LBA that does not contain the mixture parameter (or put differently, a model in which $p_{guess}$ is fixed to 0). The DIC is interpreted such that lower values signal a better trade-off between parsimony and fit to the data. For each model, we computed an overall DIC for each experiment, which aggregated across recruitment method and time-of-semester groups. We also computed the DIC for each group separately. Overall, the mixture model outperformed the standard model in both experiments (in Experiment 1, Mixture Model: 256729, Standard Model: 265245; in Experiment 2, Mixture Model: 127371, Standard Model: 128205). Of the 12 participant groups across the two experiments, the mixture model outperformed the standard model in 10 of them (full results are reported in the supplementary material). This finding suggests that in many cases the addition of the guessing process improves the ability of the model to account for the observed patterns of choices and response times.

#### Research question 1: how do method and time of recruitment relate to the proportion of guess trials, efficiency of information processing, response caution, and non-decision time?

**Fig. 5:**
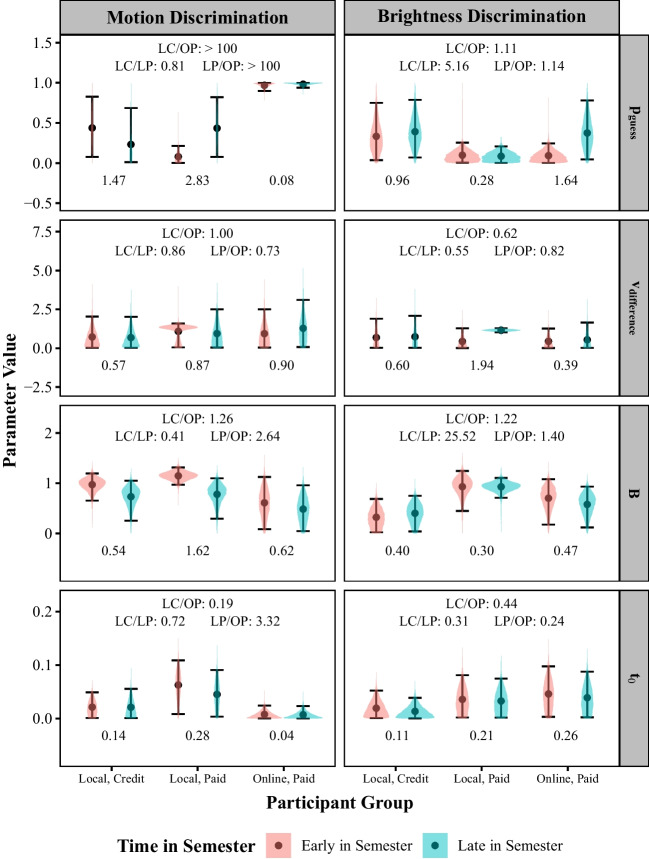
Posterior distributions on the mixture parameter ($p_{guess}$), efficiency of information processing ($v_{difference}$), response caution (*B*), and non-decision time ($t_0$) averaged across participants in each group. Points represent the mean of the distribution. Error bars span the 95% credible interval. The printed values at the top of each panel are the Bayes Factors for the comparisons of each participant population. The printed values at the bottom are the Bayes Factors testing for the time of semester effects within each participant population

The results pertaining to the first research question are presented in Fig. 5. The printed values at the top of each panel are the Bayes factors for the difference between participant populations (aggregated across times of semester). As can be seen in the top row, there was extreme evidence for a difference in guessing rates among online, paid participants compared to each of the other groups in the motion discrimination task (top-left), but not the brightness discrimination task (top-right), with the online, paid group having a higher rate of guessing than the other groups in the first experiment. There was no evidence that would be regarded as at least moderate for an effect of time-of-semester on the guessing rate within any participant population. Indeed, in the motion discrimination task, we found strong evidence for the early- versus late-in-semester online, paid groups having the same guessing rate, though this may be due to ceiling effects. We also found moderate evidence for the early- versus late-in-semester local, paid groups having the same guessing rate in the brightness discrimination task.

The second row of panels show the results for the $v_{difference}$ parameter, which reflects the efficiency of information processing. These results provide a way of assessing the expectations of the cognitive psychologists in our survey regarding the performance of the different participant groups as the rate of information processing provides an index of performance. Counter to the expectations of the cognitive psychologists, there was no evidence regarding any differences in information processing rate between participant populations in either experiment, as none of the Bayes Factors for these comparisons were greater than one. There was also no evidence for the predicted effect of time of semester among the local, credit group.

As can be seen in the third row of Fig. 5, there was strong evidence for the predicted difference in response caution between local, credit and local, paid participants in the brightness discrimination task (but not in the motion discrimination task), with local, paid participants demonstrating more caution. There was no evidence for the predicted difference in response caution between early- versus late-in-semester participants within the local, credit group in either experiment. The response caution and efficiency of information processing results together suggest that researchers’ intuitions regarding how different participants approach these tasks are not necessarily accurate.

Finally, as can be seen in the fourth row of Fig. 5, there was little evidence for differences in non-decision time among participant groups. The one exception was the moderate evidence we found for a difference between local, paid and online, paid participants in the motion discrimination task, with the former group having longer non-decision times. The Bayes factors for all other comparisons were below one. Indeed, we found moderate evidence for equivalent non-decision times between the local, credit and online, paid groups in the motion discrimination task and between the local, paid and each of the other two groups in the brightness discrimination task. We found at least moderate evidence for equivalent non-decision times between early- versus late-in-semester participants across all participant populations in both experiments.

**Fig. 6:**
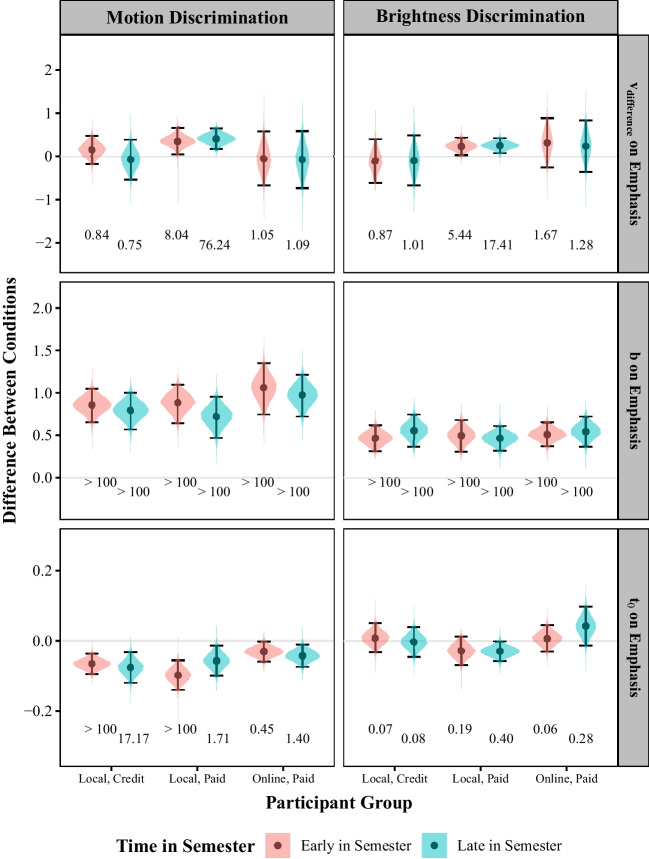
Posterior distributions representing the effects of the speed/accuracy emphasis manipulation on the efficiency of information processing ($v_{difference}$), response caution (*B*), and non-decision time ($t_0$) averaged across participants in each group. Positive values indicate that the relevant parameter was higher in the accuracy condition compared to the speed condition. Points represent the mean of the distribution. Error bars span the 95% credible interval. The printed value at the bottom are the Bayes Factors testing the null hypothesis that the relevant effect is equal to zero for that participant group

#### Research question 2: how do method and time of recruitment relate to the effects of speed/accuracy emphasis on efficiency of information processing, response caution, and non-decision time?

The results pertaining to the second research question are shown in Fig. 6. As can be seen in the top row, there was at least moderate evidence for an effect of speed/accuracy emphasis on the efficiency of information processing in each group of local, paid participants in both experiments. These participants processed information more efficiently under accuracy emphasis than under speed emphasis. As shown in the second row, there was extreme evidence for an effect of speed/accuracy emphasis on response caution in every participant group across the two experiments. Participants were more cautious when accuracy was emphasized than when speed was emphasized. Finally, in the motion discrimination task there was at least strong evidence for an effect of speed/accuracy emphasis on non-decision time among the local groups (with the exception of the late-in-semester local, paid group). These groups had shorter non-decision times under accuracy emphasis than under speed emphasis. By contrast, in the brightness discrimination task there was at least moderate evidence for the lack of an effect of speed/accuracy emphasis on non-decision time across all groups except the late-in-semester local, paid group.

#### Research question 3: how do method and time of recruitment relate to the effects of discrimination difficulty on the efficiency of information processing?

**Fig. 7:**
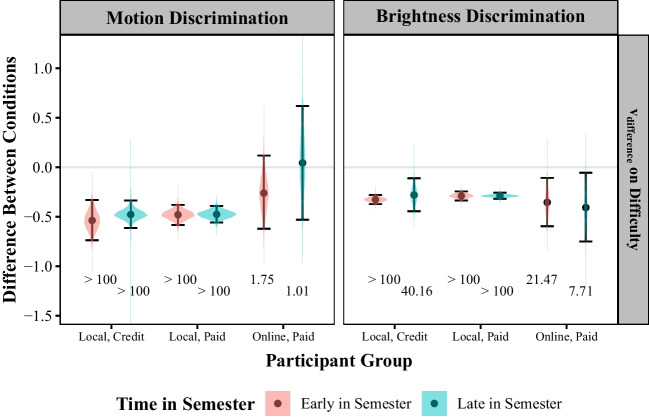
Posterior distributions representing the effects of the difficulty manipulation on the efficiency of information processing ($v_{difference}$) averaged across participants in each group. Negative coefficients indicate that the difference in rates decreased as discrimination difficulty increased. Points represent the mean of the distribution. Error bars span the 95% credible interval. The printed value at the bottom are the Bayes Factors testing the null hypothesis that the relevant effect is equal to zero for that participant group

The results pertaining to the third research question are shown in Fig. 7. In the motion discrimination task, there was at least very strong evidence for an effect of discrimination difficulty on the efficiency of information processing among the four groups of local participants (but neither of the online, paid groups). These participants processed information more efficiently as discrimination difficulty decreased. In the brightness discrimination task, there was at least moderate evidence for an effect—with information processing efficiency increasing with decreasing discrimination difficulty—in all groups.

## Discussion

Our aim was to assess the extent to which perceptual decision-making processes differ among participants recruited at different times and in different ways. We accomplished this by applying a mixture modeling approach that allowed us to quantify performance when engaged in the task, the consistency of focus across trials, response caution, and the time taken for non-decision processes, and examine how these constructs were influenced by standard manipulations of speed/accuracy emphasis and stimulus quality within the different participant groups. Our brief survey of cognitive psychologists found that researchers expected participant performance and response caution to be lower among undergraduate, course credit participants who sign up later in the semester compared with those who sign up earlier in the semester. Moreover, researchers expected local participants completing the experiment in the lab in exchange for financial remuneration to both perform better and be more cautious than undergraduate course credit participants and participants recruited via online crowd-sourcing platforms.

We then reported two experiments examining whether the above expectations are reflected in participant data. Using two common perceptual decision-making tasks – a motion discrimination task in Experiment 1 and a brightness discrimination task in Experiment 2 – we examined whether responses to standard manipulations of discrimination difficulty and speed/accuracy emphasis differed across the participant groups. Counter to the expectations set by our survey, across both experiments, course credit participants recruited later in semester were no more accurate or faster to respond than those recruited earlier in semester. In the motion discrimination task, it was actually the paid participants who were more accurate when recruited earlier in semester, whereas course credit participants were actually more accurate when recruited later in semester. Among the course credit participants, the mixture modeling revealed very little evidence for time-of-semester effects on performance when engaged in the task (as measured by the difference in drift rate parameters of the LBA for the evidence accumulators corresponding to the correct and incorrect responses), consistency in task focus (as measured by the mixture parameter), response caution (as measured by the threshold parameter), and non-decision time.

In both experiments, local participants who completed the experiment in exchange for financial remuneration were the most accurate of the three groups. However, counter to the expectations set by our survey, the mixture modeling revealed little evidence for differences in performance when engaged in the task among participants recruited in different ways. The evidence for differences in caution among these participant groups was mixed. In the brightness discrimination task, as predicted by our survey respondents, local participants completing the experiment in the lab in exchange for financial remuneration responded more cautiously than course credit participants. However, little evidence emerged for other differences between the three recruitment groups.

The course credit participants who signed up early versus late in the semester both showed decreases in information processing efficiency when stimuli were harder to discriminate. The two groups also both showed increases in response caution when accuracy was emphasized. However, we found evidence that the effects of the experimental manipulations may differ across participant populations. In the motion discrimination task, the effects of speed/accuracy emphasis and discrimination difficulty on accuracy were both strongest among the local participants (though this may have been due to the floor effects on accuracy among the online participants in this task). By contrast, the effect of speed/accuracy emphasis on response time was strongest among the online participants (as well as local, paid participants in the brightness discrimination task). The mixture modeling suggested that when accuracy was emphasized, all groups were more cautious, but only the local, paid participants showed evidence of increased performance. Moreover, the local groups in the motion discrimination task showed evidence of decreased non-decision time when accuracy was emphasized, but this was not the case for the online participants in the motion discrimination task or any of the groups in the brightness discrimination task. Indeed, in the brightness task, the evidence suggested that non decision-time was the same between emphasis conditions for most groups. Finally, all participant groups except the online, paid groups in the motion discrimination task demonstrated reduced efficiency of information processing in response to increasing discrimination difficulty.

One particularly interesting finding was the high rate of guessing responses among the online participants in the motion discrimination task. In this task, the online participants performed only slightly better than chance. By contrast, participants who completed the brightness discrimination task online performed at about the same level as the local participants. The fact that online participants were classified as guessing more in the motion discrimination task but not the brightness discrimination task suggests that the poor performance in the former task is not due to a general lack of motivation among online participants. We believe this difference is more likely attributable to differences in the experimental stimuli used in the two tasks. The motion discrimination task used a random dot kinematogram stimulus, which requires dots to appear and disappear from the screen at precisely timed intervals in order to give the appearance of motion. It is possible that certain online participants completed the experiment using devices on which the random dot stimulus did not render cleanly, making it more difficult to discern the direction of motion. The brightness discrimination task, by contrast, used a static stimulus that did not require the same precision in timing in order to render. As such, the presentation of this stimulus may have been less affected by the device used to complete the experiment. These potential limitations in how stimuli are displayed to online participants are consistent with the issues raised by Crump et al. (2013) in their attempt to replicate other cognitive experiments using participants recruited via Amazon Mechanical Turk.

### Recommendations

On the basis of these findings, we believe that undergraduate participants completing a simple, perceptual experiment in exchange for course credit are likely to provide similar patterns of results regardless of whether they are recruited early versus late in the semester. At least for tasks such as the ones reported here, we do not believe researchers need to be concerned about substantial decreases in data quality over the academic term.

We also believe that participants recruited from online platforms such as Amazon Mechanical Turk can provide perceptual decision-making data that is at least similar in quality to undergraduate course credit or paid participants who complete the experiment in the lab. However, in order to adequately account for the propensity of participants to guess on certain trials, we recommend using the mixture modeling approach demonstrated here. We also recommend that the experiment be rigorously piloted tested to ensure that the experimental stimuli are appropriate for the devices participants are using to complete the experiment. Where possible, using tasks with static stimuli such as brightness or numerosity judgment tasks instead of tasks with more dynamic stimuli such as dot motion discrimination may help to reduce noise and improve data quality.

It is important that the reader be reminded that our online sample was limited to Mechanical Turk workers with approval rates of 95% or more who were compensated at a rate similar to our in-lab participants ($4 USD). We cannot be sure that our conclusions hold for workers with lower approval rates or who are paid less. It is possible that those with lower approval rates or who receive less compensation may be less attentive, less responsive to experimental manipulations, or less motivated when performing the task. Our endorsement, therefore, does not extend to online samples that differ from ours on these dimensions. Researchers wishing to use participants with lower approval rates or different levels of compensation will need to conduct further testing to examine whether there are systematic differences between the participants who enroll under those conditions and the ones who enrolled in our study.

### Generalizability and future work

It’s important to note that although we observed differences between the three participant groups, there are still unanswered questions about why these differences emerged. One question is whether these group differences were due to who the participants were (undergraduate students, members of the local community, or online workers), how they were compensated (money versus course credit), or where they completed the study (online versus in the lab). These factors were conflated in our study, making it difficult to infer, for example, the extent to which the behavior of the undergraduate group was due to their being undergraduates or their being awarded course credit. In some cases, as in the previous example, the conflation of certain factors seems hard to avoid. However, the recent increase in online testing of undergraduate students who participate for course credit provides an opportunity for at least some of these factors to be better disentangled in future work. For example, by comparing undergraduate students participating in the lab versus online, future work might be better able to isolate the influence of where the study is completed.

Another open question is how much the group differences we observed are attributable to the characteristics of the participants within each group rather than how they were compensated or where they completed the study. We collected age and gender data in this study, which indeed differed between groups—the Mechanical Turk workers tended to be older and were more likely to be male. Other work has shown that these participants differ on a range of personality variables from participants recruited from more traditional means (e.g., undergraduates, clinical populations, Miller et al., 2017). It is possible that one or more of these individual difference variables contributes to the effects observed here. We, therefore, urge caution in generalizing these results to other contexts where these variables may differ, even if the same participants are being recruited from the same source.

It may also be the case that participants from other crowdsourcing platforms can provide higher-quality data than Mechanical Turk workers with high approval rates. Hauser et al. (2022) showed that even when requiring a 95% approval rate, participants recruited through the CloudResearch platform showed better attention and comprehension of instructions, and a lower propensity to cheat, than participants recruited through Mechanical Turk. It is therefore as of yet unclear whether the effects observed among the online-paid participants generalize to other platforms.

Opportunities also exist to develop more sophisticated accounts of the patterns of non-stimulus-driven responses. Our model was agnostic to the temporal ordering of guess responses, effectively assuming that guess trials are independently distributed. A more detailed specification of this process might assume the existence of a higher-order ”guessing state” which persists for a string of consecutive trials producing runs of guess responses (e.g., Ashwood et al., 2022). This would be an especially useful way of examining factors that predict the likelihood of guess responses (e.g., task fatigue).

A final question is whether the differences we observed generalize across tasks. The two paradigms we used in this research—the random dot and brightness discrimination tasks—are quite similar in terms of the cognitive processes they are thought to engage. Yet we still found differences between the tasks with regard to certain effects. A useful next step then would be to examine whether other, more complex cognitive tasks thought to engage the evidence accumulation process, such as conflict, learning, or inhibition tasks, elicit different patterns of results.

Open Practices Statement: The data and code necessary to conduct all the analyses presented in this paper, as well as the source code used to generate the experimental stimuli, are publicly available, and can be found at https://osf.io/qgzfw/. All studies presented in this paper were preregistered. The preregistrations can be found at https://osf.io/rax2b.

## Footnotes

1 As a result, correct guesses will be slower on average than correct stimulus-driven responses, but incorrect guesses will be faster than incorrect stimulus-driven responses.

2 Note that the mixture component of the LBA model was not included in our preregistration.

3 The posterior distributions were estimated using the differential evolution Markov chain Monte Carlo (DE-MCMC) algorithm (Turner et al., 2013; Ter Braak, 2006), which involves auto-tuning the proposed parameter values for each chain on each iteration using the difference in parameter values between two other randomly sampled chains. Importantly, the DE-MCMC algorithm is able to efficiently move through highly-correlated parameter spaces, which are common in models of response time (Turner et al., 2013). For each model we used $3 \times k$ parallel chains that ran for 5,000 iterations, where *k* is the number of free parameters per participant in the model, and the first 2,000 iterations were discarded as burn-in.
